# Multiscale-omic assessment of *EWSR1-NFATc2* fusion positive sarcomas identifies the mTOR pathway as a potential therapeutic target

**DOI:** 10.1038/s41698-021-00177-0

**Published:** 2021-05-21

**Authors:** Nathan D. Seligson, Richard D. Maradiaga, Colin M. Stets, Howard M. Katzenstein, Sherri Z. Millis, Alan Rogers, John L. Hays, James L. Chen

**Affiliations:** 1 Department of Pharmacotherapy and Translational Research, The University of Florida, Jacksonville, FL USA; 2 Department of Pharmacogenomics and Translational Research, Nemours Children’s Specialty Care, Jacksonville, FL USA; 3 Division of Pediatric Hematology/Oncology, Department of Pediatrics, Nemours Children’s Specialty Care, Jacksonville, FL USA; 4 The Ohio State University Wexner Medical Center and Comprehensive Cancer Center, The Ohio State University, Columbus, OH USA; 5 Foundation Medicine Inc, Cambridge, USA; 6 Department of Radiology, The Ohio State University, Columbus, OH USA; 7 Division of Medical Oncology, Department of Internal Medicine, The Ohio State University, Columbus, OH USA; 8 Division of Gynecologic Oncology, Department of Obstetrics and Gynecology, The Ohio State University, Columbus, OH USA; 9 Department of Biomedical Informatics, The Ohio State University, Columbus, OH USA

**Keywords:** Predictive markers, Sarcoma, Tumour biomarkers

## Abstract

Sarcomas harboring *EWSR1-NFATc2* fusions have historically been categorized and treated as Ewing sarcoma. Emerging evidence suggests unique molecular characteristics and chemotherapy sensitivities in *EWSR1-NFATc2* fusion positive sarcomas. Comprehensive genomic profiles of 1024 *EWSR1* fusion positive sarcomas, including 14 *EWSR1-NFATc2* fusions, were identified in the FoundationCore® database. Additional data from the Gene Expression Omnibus, the Genomics of Drug Sensitivity in Cancer and The Cancer Genome Atlas datasets were included for analysis. *EWSR1-NFATc2* fusion positive sarcomas were genomically distinct from traditional Ewing sarcoma and demonstrated upregulation of the mTOR pathway. We also present a case of a 58-year-old male patient with metastatic *EWSR1-NFATc2* fusion positive sarcoma who achieved 47 months of disease stabilization when treated with combination mTOR and VEGF inhibition. *EWSR1-NFATc2* fusion positive sarcomas are molecularly distinct entities with overactive mTOR signaling; which may be therapeutically targetable. These findings support the use of precision medicine in the Ewing family of tumors.

## Introduction

The Ewing gene (EWS RNA Binding Protein 1; *EWSR1*) is commonly identified in soft-tissue sarcomas (STS) as an oncogenic fusion gene associated with a number of partner genes^1^. *EWSR1* fusions have been identified in a number of STS subtypes, including Ewing sarcomas, desmoplastic small round cell tumors, and mesenchymal chondrosarcomas^2^. Fusion partners for *EWSR1* have most commonly been identified as belonging to the *ETS* family of genes, notably *FLI1* and *ERG*; however, a more recently described fusion partner for the *EWSR1* gene, Nuclear Factor of Activated T cells cytoplasmic 2 (*NFATc2, NFAT1, or NFATp*), is estimated to constitute 4–6% of EWSR1 fusions^3–5^. Historically, *EWSR1-NFATc2* fusion positive sarcomas have been classified as a member of the Ewing family of sarcomas; however, growing evidence suggests that *EWSR1-NFATc2* fusion positive sarcomas should be considered separate from standard Ewing sarcomas^5–14^.

*NFATc2* is part of a family of transcription factors responsible for T-cell differentiation and cytokine activation^15,16^. Genomic variants in *NFATc2* have been described in the pathogenesis of solid and hematologic malignancies. Dysregulation of *NFATc2* is thought to be involved in multiple biologic mechanisms including induction of tumor invasion^17,18^, repression of tumor suppressor genes^19,20^, and tumor-induced T-cell anergy^21^. Little data exists to guide the clinical care of *EWSR1-NFATc2* fusion positive sarcomas due to their rarity. Currently, only 12 case reports (Supplementary Table 1), corresponding to six unique patients, describe clinical outcomes following systemic chemotherapy in *EWSR1-NFATc2* fusion positive sarcomas^5–7,22–25^. The cases reported were mostly seen in males with an age ranging from 12 to 58 years at diagnosis and were commonly seen in the extremities. Of the patients who received traditional cytotoxic therapy for Ewing sarcoma, few derived meaningful benefit from initial treatment.

Molecularly, *EWSR1-NFATc2* fusion positive sarcomas are distinct from other *EWSR1* fusion positive sarcomas as measured by gene expression and methylation profiles^26–28^. Further assessment of the molecular differences between *EWSR1-NFATc2* and other *EWSR1* fusion positive sarcomas may shed light on the disparity of response to chemotherapeutics while providing insight into new treatment methods for these patients. Here we present the largest cohort of genomically profiled *EWSR1-NFATc2* fusion positive sarcomas and assess secondary alterations associated with the fusion relative to other *EWSR1* fusion sarcomas. Additionally, we evaluate altered pathways using transcriptomic data and highlight the importance of the mTOR pathway in *EWSR1-NFATc2* fusion positive sarcomas. Finally, we present a case of a long-term responder to everolimus-based therapy.

## Results

### EWSR1-NFATc2 fusion positive sarcoma subject demographics

Fourteen subjects with *EWSR1-NFATc2* fusion positive sarcoma were identified in the Foundation Medicine FoundationCore^®^ (FMI) research database from a total of 1024 *EWSR1* fusions, resulting in an overall prevalence of 1.4% among *EWSR1* fusion positive sarcomas. A majority of the patients were male (9, 63.6%) with a median age of 40.9 (range, 20–70) at time of diagnosis. Reported histology for these samples was most commonly identified as soft tissue Ewing sarcoma (4, 28.6%) and soft tissue sarcoma not otherwise specified (3, 21.4%). No other histology was identified in more than one sample. Full demographic data is available in Table 1.

**Table 1:** Demographics of *EWSR1-NFATc2* samples.

*EWSR1-NFATc2* samples *n* = 14	
*Sex*	
Female	5 (35.7%)
Male	9 (64.3%)
Age at sequencing (mean [SD])	40.1 [14.8] years
*MSI status*	
Not performed	5
Stable	9
Tumor mutation burden (TMB, mean [SD])	1.5 [1.7] mutations/megabase
*Known secondary pathogenic variants*	
0	3
1	4
2	4
≥3	3
*Histology*	
Soft tissue ewing sarcoma	4 (28.6%)
Soft tissue sarcoma (nos)	3 (21.4%)
Bone chondrosarcoma	1 (7.1%)
Bone osteosarcoma	1 (7.1%)
Soft tissue chondrosarcoma	1 (7.1%)
Soft tissue hemangioma	1 (7.1%)
Soft tissue malignant peripheral nerve sheath tumor (mpnst)	1 (7.1%)
Soft tissue myoepithelial carcinoma	1 (7.1%)
Soft tissue round cell tumor	1 (7.1%)

Gene names are shown in italics.

### EWSR1-NFATc2 positive sarcomas demonstrate recurrent fusion breakpoints

Fusion breakpoints for the *EWSR1-NFATc2* fusion positive samples identified in the FMI research database, in regard to the *NFATc2* gene, demonstrated consistent breakpoints on *NFATc2* for each of the 14 samples. In seven samples (50%) the breakpoint for the *NFATc2* gene was located between exons 2 and 3, while the other seven samples (50%) exhibited breakpoints within exon 3 of *NFATc2* (Table 2). This is consistent with previous reports suggesting that the primary transactivation domain and regulatory domains of *NFATc2* are conserved in the *EWSR1-NFATc2* fusion^5^. Similarly, breakpoints on *EWSR1* were consistent with previous findings implicating exons 6, 7, 8, 9, and 10 as common breakpoint regions^1,29^.

**Table 2:** Detected breakpoints in *EWSR1-NFATc2* fusion.

Case #	*EWSR1* (5’ Breakpoint)			*NFATc2* (3’ Breakpoint)		
	Chromosome	Breakpoint		Chromosome	Breakpoint	
		Base	Exon		Base	Exon
1	22	29,687,407	Between Exon 8 and Exon 9	20	50,138,336	Between Exon 2 and Exon 3
2	22	29,678,464	Exon 6	20	50,133,377	Exon 3
3	22	29,684,740	Exon 8	20	50,133,396	Exon 3
4	22	29,684,715	Exon 8	20	50,133,388	Exon 3
5	22	29,687,841	Between Exon 9 and Exon 10	20	50,136,578	Between Exon 2 and Exon 3
6	22	29,688,045	Between Exon 9 and Exon 10	20	50,138,056	Between Exon 2 and Exon 3
7	22	29,687,630	Between Exon 9 and Exon 10	20	50,134,569	Between Exon 2 and Exon 3
8	22	29,685,425	Between Exon 8 and Exon 9	20	50,134,374	Between Exon 2 and Exon 3
9	22	29,684,680	Exon 8	20	50,133,375	Exon 3
10	22	29,687,615	Between Exon 9 and Exon 10	20	50,136,745	Between Exon 2 and Exon 3
11	22	29,678,508	Exon 6	20	50,133,388	Exon 3
12	22	29,685,701	Between Exon 8 and Exon 9	20	50,135,097	Between Exon 2 and Exon 3
13	22	29,684,680	Exon 8	20	50,133,379	Exon 3
14	22	29,684,695	Exon 8	20	50,133,380	Exon 3

Gene names are noted in italics.

### The secondary genomic landscape identifies EWSR1-NFATc2 fusion positive sarcomas as distinct from classical Ewing sarcoma

**Fig. 1:**
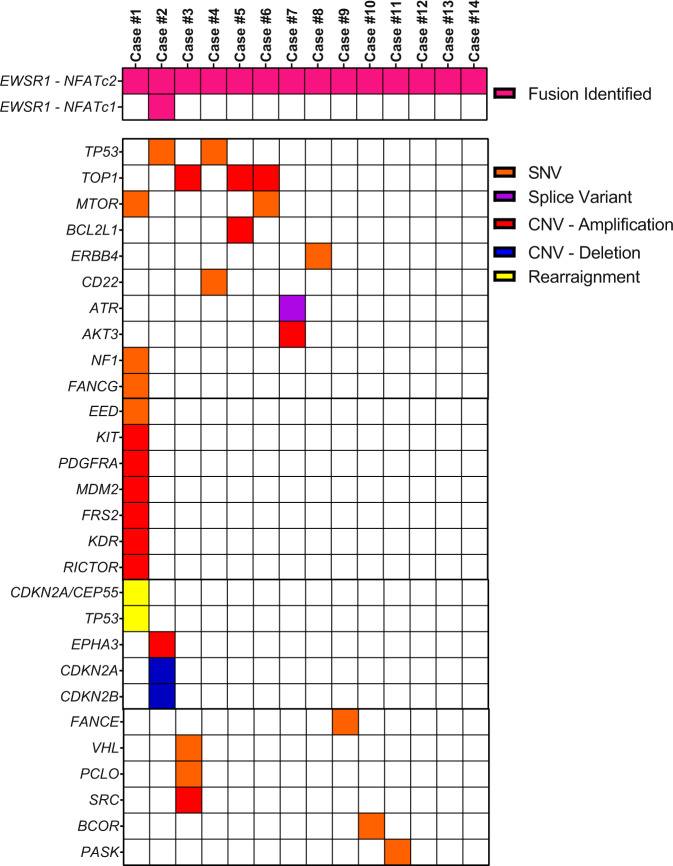
Genomic landscape of *EWSR1-NFATc2* fusion positive sarcomas. Heatmap of the presence or absence of known pathogenic genomic variants in 14 subjects with *EWSR1-NFATc2* fusion positive sarcoma identified in the FMI research database. The *EWSR1-NFATc2* fusion was identified as the only pathogenic genomic variant in three subjects (27.3%), while one subject exhibited both an *EWSR1-NFATc2* and *EWSR1-NFATc1* fusion simultaneously. Secondary pathogenic genomic variants were identified in *TOP1*, *TP53*, *BCOR*, *AKT3*, *ATR*, *BCL2L1*, *CD22*, *CDKN2A, CDKN2B*, *EPHA3*, *ERBB4*, *FANCE*, and *mTOR*. Only three genes, *mTOR, TOP1*, and *TP53*, exhibited pathogenic variants in more than one subject. SNV single nucleotide variant, CNV copy number variant.

*EWSR1-NFATc2* fusion positive sarcomas exhibited a relatively stable genome. The *EWSR1-NFATc2* fusion was identified as the only pathogenic genomic variant in three patients (27.3%). Of note, one subject exhibited both an *EWSR1-NFATc2* and *EWSR1-NFATc1* fusion simultaneously. Pathogenic genomic variants were identified in 27 unique genes (Fig. 1; Supplementary Table 2). Only three genes, *TOP1, mTOR*, and *TP53*, exhibited pathogenic variants in more than one sample.

We then compared the secondary genomic landscape of *EWSR1-NFATc2* fusion positive sarcomas to other classical Ewing and Ewing-family variant samples from the FMI database. Partner genes to *EWSR1* were included if there were a minimum of 10 samples with the given fusion available for analysis. In addition to the 14 *EWSR1-NFATc2* positive sarcomas, 1010 other *EWSR1* fusion positive sarcomas were identified in the FMI database including 447 *EWSR1-FLI1*; 159 *EWSR1-ATF1*; 151 *EWSR1-WT1*; 88 *EWSR1-NR4A3*; 49 *EWSR1–ERG*; 46 *EWSR1-CREB3L1*; 29 *EWSR1-CREB1*; 15 *EWSR1-CREM*; 15 *EWSR1-PATZ1*; and 11 *EWSR1-CREB3L1* positive sarcomas.

**Fig. 2:**
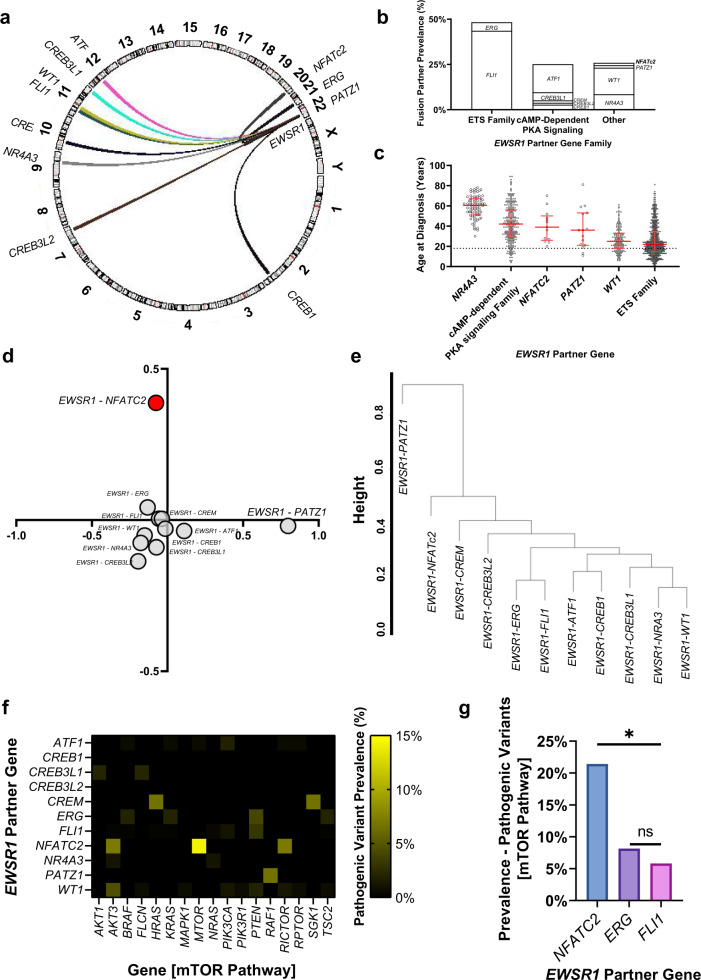
Genomic landscape of *EWSR1* fusion positive sarcomas identifies unique properties of *EWSR1-NFATc2* positive sarcomas. Genomic data from the FoundationCore research database identified 1024 *EWSR1* fusion positive sarcomas, including 447 *EWSR1-FLI1*; 159 *EWSR1-ATF1*; 151 *EWSR1-WT1*; 88 *EWSR1-NR4A3*; 49 *EWSR1–ERG*; 46 *EWSR1-CREB3L1*; 29 *EWSR1-CREB1*; 15 *EWSR1-CREM*; 15 *EWSR1-PATZ1*; 11 *EWSR1-CREB3L1;* and 14 *EWSR1-NFATc2* positive sarcomas. **a** and **b** Assessment of partner genes in *EWSR1* fusion positive sarcomas found no preference for specific chromosomes. Fusion partners with *EWSR1* are represented in a circus plot with connecting line width representing fusions identified within the dataset. Fusion partners were most prevalent on chromosome 11, driven by *FLI1* (**a**). All partner genes identified were transcription factors; most of which were identified as members of the ETS family or involved in cAMP-dependent PKA signaling (**b**). **c** *EWSR1* fusion positive sarcomas whose partner genes were not members of the ETS family were seen in significantly older patients with the exception of *EWSR1-WT1* positive sarcomas (age at diagnosis [mean ± SD, *p*-value comparison to *EWSR1-*ETS by one-way ANOVA]: *EWSR1-*ETS 25.8 ± 15.8, *p* = NA; *EWSR1-WT1* 26.9 ± 12.2, *p* = 0.9; *EWSR1-PATZ1* 37.1 ± 20.1, *p* = 0.03; *EWSR1-NFATc2* 40.1 ± 15.4, *p* = 0.006; *EWSR1-* AMP-dependent PKA signaling Family 42.5 ± 18.3, *p* < 0.0001; *EWSR1-NR4A3* 58.7 ± 10.9, *p* < 0.0001). **d** and **e** Principle component analysis identified similarity between secondary genomic landscapes of *EWSR1* fusion positive sarcomas. *EWSR1-NFATc2* positive sarcomas represent a genomically distinct subset of *EWSR1* fusion positive sarcomas (**d**). With the exception of *EWSR1-NFATc2* and *EWSR1-PATZ1* positive sarcomas, most fusion subtypes clustered together. **e** Dendrogram representing hierarchical clustering of *EWSR1* fusion positive sarcomas by fusion partner gene. **f** Prevalence of pathogenic variants in the mTOR pathway, defined by KEGG by fusion partner gene. **g** Comparing the frequency of known pathogenic genomic variants in the mTOR pathway, defined by KEGG, in *EWSR1-NFATc2, EWSR1-FLI1*, and *EWSR1–ERG* fusion positive samples, identified an enrichment of mTOR variants in *EWSR1-NFATc2* fusion positive samples (*EWSR1-NFATc2* 21.4%*, EWSR1-FLI1* 5.8%*, EWSR1–ERG* 8.2%; *p* = 0.05). n.s. not significant; *p ≤ 0.05.

Assessment of partner genes in *EWSR1* fusion positive sarcomas found that fusion partner genes were most prevalent on chromosome 11, driven by *FLI1* (Fig. 2a). All partner genes identified were transcription factors; most of which were identified as members of the ETS family or those involved in cAMP-dependent PKA signaling (Fig. 2b). Taken together, this may suggest no preference of the *EWSR1* fusion for specific chromosomal locus, but for transcription factors alone in terms of its oncogenic potential. Additionally, *EWSR1* fusion positive sarcomas whose partner genes were not members of the ETS family were seen in significantly older patients with the exception of *EWSR1-WT1* positive sarcomas (age at diagnosis (years) [mean ± SD, *p*-value comparison to *EWSR1-*ETS by one-way ANOVA]: *EWSR1-*ETS 25.8 ± 15.8, *p* = NA; *EWSR1-WT1* 26.9 ± 12.2, *p* = 0.9; *EWSR1-PATZ1* 37.1 ± 20.1, *p* = 0.03; *EWSR1-NFATc2* 40.1 ± 15.4, *p* = 0.006; *EWSR1-* AMP-dependent PKA signaling Family 42.5 ± 18.3, *p* < 0.0001; *EWSR1-NR4A3* 58.7 ± 10.9, *p* < 0.0001; Fig. 2c).

Gene alteration frequency for each fusion partner of *EWSR1* in the FMI dataset was assessed by principle component analysis (PCA; Fig. 2d). While standard Ewing sarcoma fusions, *EWSR1-FLI1* and *EWSR1-ERG*, clustered tightly, *EWSR1-NFATc2* and *EWSR1-PATZ1* fusions defined independent clusters. Assessment of gene contribution to the PCA analysis showed *CDKN2A/B* variants in *EWSR1-PATZ1* fusion samples as previously reported^30^. In contrast, *TOP1*, TP53, *NF1*, and *mTOR* variants helped define the *EWSR1-NFATc2* fusion positive samples (Supplementary Fig. 1). A second independent clustering methodology (hierarchical clustering using the Euclidian distance) also demonstrated *EWSR1-FLI1* and *EWSR1-ERG* as highly similar, while *EWSR1-NFATc2* did not cluster well with any other fusion partner (Fig. 2e).

*STAG2*, a gene commonly altered in Ewing sarcoma and associated with a poor prognosis^31,32^, was not altered in a single subject with *EWSR1-NFATc2* positive sarcoma. To test the statistical difference between the genomic landscape of *EWSR1-FLI1/ERG* and *EWSR1-NFATc2* positive sarcomas, *EWSR1-FLI1* and *EWSR1–ERG* gene variant frequencies were combined. Chi-squared analysis demonstrated a statistical difference between *EWSR1-FLI1/ERG* and *EWSR1-NFATc2* positive sarcomas across all genes included in the dataset (*p* < 0.001).

### mTOR signaling is strongly associated with the EWSR1-NFATc2 fusion

*EWSR1-NFATc2* fusion positive sarcomas demonstrate a unique secondary genomic profile from other Ewing sarcomas driven by mTOR signaling. Genes associated with the mTOR pathway here were assigned based on the Kyoto Encyclopedia of Genes and Genomes (KEGG)^33–35^. Comparing the frequency of mTOR pathway known pathogenic genomic variants in *EWSR1-NFATc2, EWSR1-FLI1*, and *EWSR1–ERG* fusion positive samples identified an enrichment of mTOR variants in *EWSR1-NFATc2* fusion positive samples (*EWSR1-NFATc2* 21.4%*, EWSR1-FLI1* 5.8%, and *EWSR1–ERG* 8.2%; *p* = 0.05; Fig. 2f, g).

Previous study of gene expression identified *EWSR1-NFATc2* fusion positive sarcomas to be distinct from classic Ewing sarcoma^26^. As an independent assessment of the biologic activity of *EWSR1-NFATc2* positive sarcomas in comparison to other Ewing and Ewing family sarcomas, we interrogated two gene expression datasets consisting of 7 *EWSR1-NFATc2* positive sarcomas, 14 *CIC-DUX4* positive sarcomas, and *117 EWSR1-ETS* positive sarcomas downloaded from the GEO database. In these samples *NFATc2* was significantly overexpressed in *EWSR1-NFATc2* positive sarcomas (mean ± SD: *EWSR1-NFATc2* 11.3 ± 0.6; *CIC-DUX4* 5.7 ± 0.9; *EWSR1-ETS* 5.9 ± 1.1; *EWSR1-NFATc2* vs *EWSR1-ETS* *p* < 0.0001; *EWSR1-NFATc2* vs. *CIC-DUX4* *p* < 0.0001; *CIC-DUX4* vs. *EWSR1-ETS* *p* = 0.34; Supplementary Fig. 2A). *CD99*, a traditional cell surface marker associated with Ewing sarcoma, was equally expressed in *EWSR1-NFATc2* compared to *EWSR1-ETS* positive sarcomas, but lowly expressed in *CIC-DUX4* positive sarcomas (mean ± SD: *EWSR1-NFATc2* 12.3 ± 0.6; *CIC-DUX4* 11.2 ± 1.0; *EWSR1-ETS* 12.3 ± 0.4; *EWSR1-NFATc2* vs. *EWSR1-ETS* *p* = 0.81; *EWSR1-NFATc2* vs. *CIC-DUX4* *p* = 0.02; *CIC-DUX4* vs. *EWSR1-ETS* *p* < 0.0001; Supplementary Fig. 2B). Pathway analysis identified a significant activation of the mTOR pathway in *EWSR1-NFATc2* positive sarcomas compared to either *EWSR1-ETS* or *CIC-DUX4* positive sarcomas (*EWSR1-NFATc2* vs. *EWSR1-ETS*: *z*-score = 1.6, *p* = 0.002; *EWSR1-NFATc2* vs. *CIC-DUX4*: *z*-score = 1, *p* < 0.001; Supplementary Fig. 2C, D). No discernable difference in mTOR pathway activation was identified between *EWSR1-ETS* and *CIC-DUX4* positive sarcomas (*EWSR1-ETS* vs. *CIC-DUX4*: *z*-score = −0.2, *p* = 0.01).

### In vitro database analysis supports biologic connection between NFATc2 and the mTOR pathway

**Fig. 3:**
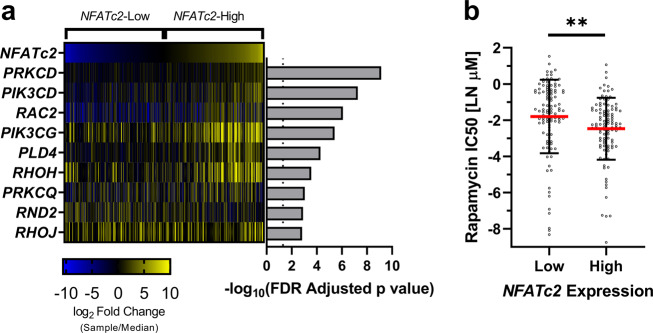
In vitro data identifies relationship between *NFATc2* expression and the mTOR pathway. Data from the Genomics of Drug Sensitivity in Cancer database, including DNA variants, mRNA expression, and drug sensitivity, were collected for 1002 cell lines. To reduce the effect of DNA level variants on the mTOR pathway, cell lines identified to have known cancer driver genomic variants in *PTEN*, *mTOR*, *TSC1*, or *TSC2* were excluded from our analysis. Cell lines were divided into *NFATc2*-Low and *NFATc2*-High categories by median *NFATc2* expression. Differential expression was followed by pathway analysis. *NFATc2*-High lines exhibited activation of the mTOR pathway (*z*-score = 5.0, *p* < 0.0001). **a** Heatmap of mTOR pathway genes from pathway analysis identified to be differentially expressed between *NFATc2*-Low and *NFATc2*-High cell lines. **b** *NFATc2*-High lines were more sensitive to the mTOR inhibitor rapamycin (mean ± SD: *NFATc2*-Low −1.8 ± 2.0, *NFATc2*-High −2.5 ± 1.7, *p* = 0.009). Plot represent the center line as the mean, error bars as ±SD. ns not significant; *p ≤ 0.05; **p < 0.01; ***p < 0.001.

Data from the Genomics of Drug Sensitivity in Cancer database and the Broad Institute Cancer Cell Line Encyclopedia (CCLE), including DNA variants, mRNA expression, and drug sensitivity, were collected for 935 cancer cell lines. To reduce the effect of activating/silencing gene mutations on mTOR transcriptomic analysis, cell lines with cancer driving genomic alterations in *PTEN*, *mTOR*, *AKT1*, *AKT2*, *AKT3*, *PI3KCA, TSC1*, or *TSC2* were excluded. Cell lines were divided into *NFATc2*-Low and *NFATc2*-High categories by median *NFATc2* expression. Differential expression was followed by pathway analysis. *NFATc2*-High cell lines exhibited activation of the mTOR pathway (*z*-score = 4.8, *p* < 0.0001). Assessment of genes contributing to mTOR activation of the mTOR pathway in *NFATc2*-High cell lines identified significant differential expression in 9 of 93 genes included in the IPA mTOR pathway (Gene[log2 fold change *NFATc2*-High/*NFATc2*-Low, false discovery rate (FDR) adjusted *p*-value]; *PRKCD* [2.1, 7.4 × 10^−10^]; *PIK3CD* [1.6, 5.4 × 10^−8^]; *RAC2* [1.3, 8.4 × 10^−7^]; *PIK3CG* [2.0, 3.8 × 10^−6^]; *PLD4* [2.9, 5.1 × 10^−5^]; *RHOH* [1.4, 2.8 × 10^−4^]; *PRKCQ* [1.2, 9.1 × 10^−4^]; *RND2* [−1.2, 1.2 × 10^−3^]; *RHOJ* [1.3, 1.5 × 10^−3^]; Fig. 3a). *NFATc2*-High cell lines were more sensitive to the mTOR inhibitor rapamycin (LN IC_50_ µM Mean ± SD: *NFATc2*-Low −1.8 ± 2.0, *NFATc2*-High −2.5 ± 1.7, *p* = 0.009**;** Fig. 3b).

### Pan-cancer analysis identifies NFATc2 mRNA expression as a potential mTOR pathway driver

To further assess the potential biologic correlation between *NFATc2* mRNA expression and the mTOR pathway, data from 33 non-overlapping TCGA datasets were selected for analysis. *NFATc2* genomic amplification was poorly correlated with mRNA expression across TCGA datasets (Supplementary Fig. 3a–c). To assess the effect of *NFATc2* mRNA expression on cancer biology, *NFATc2*-High and *NFATc2*-Low expressing samples were selected for differential expression and pathway analysis from each dataset (Supplementary Table 3).

**Fig. 4:**
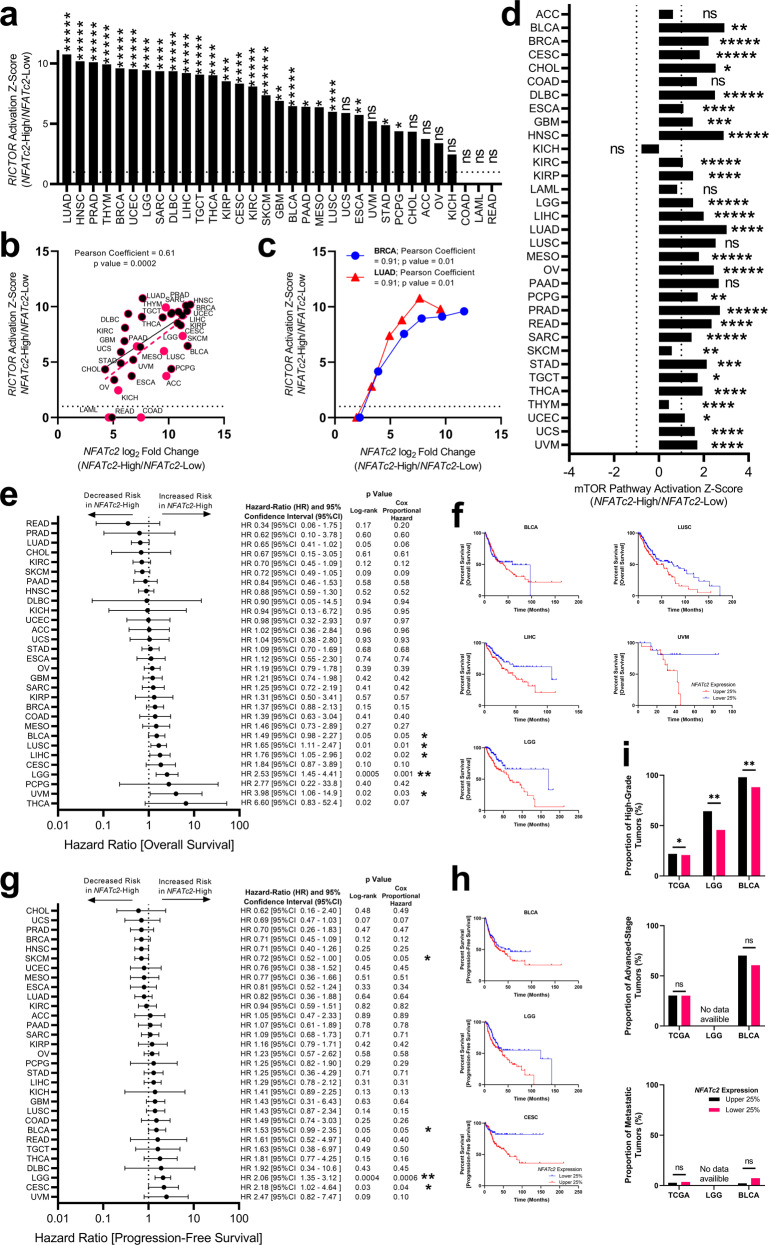
TCGA Pan-cancer analysis suggests correlation between *NFATc2* mRNA expression and mTOR pathway activation. Genomic data from 33 non-overlapping TCGA datasets, including 10,124 total tumor samples, were downloaded from firebrowse.org^57^. To assess for the effect of extreme *NFATc2* expression, high and low *NFATc2* expressing samples were selected for each dataset (*NFATc2-*High and *NFATc2-*Low; Supplementary Table 3). Once the percentile cutoffs were identified for each dataset, differential expression and pathway analysis (IPA) was conducted. **a** Twenty-four (72.7%) datasets exhibited a statistically significant activation of *RICTOR* in *NFATc2*-High samples. **b** When considering each dataset, the magnitude of the difference in *NFATc2* expression between *NFATc2*-High and *NFATc2*-Low samples was highly correlated with the estimated activation of *RICTOR* (Pearson coefficient = 0.61, *p* = 0.0002). Black-filled circles represent datasets where mTOR was significantly activated in *NFATc2*-High samples. **c** For the largest TCGA dataset, BRCA, and the dataset with the most activated *RICTOR* signal, LUAD, high and low *NFATc2* expressing samples were selected at multiple percentile cutoffs (Supplementary Table 4). For both BRCA and LUAD datasets, the magnitude of the difference in *NFATc2* expression between *NFATc2*-High and *NFATc2*-Low samples was highly correlated with the estimated activation of *RICTOR* (BRCA: Pearson coefficient = 0.91, *p* = 0.01; LUAD: Pearson coefficient = 0.91, *p* = 0.01). **d** Twenty-five (75.5%) datasets exhibited significant upregulation of mTOR pathway in *NFATc2-*High samples. **e–i** For clinical analysis of samples with high or low *NFATc2*, mRNA expression was selected for each dataset in the top and bottom quartile of *NFATc2* expression (Supplementary Table 5). **e** Forest plot of association between *NFATc2*-High or *NFATc2*-Low expressers and overall survival for 30 datasets with valid outcomes data. *NFATc2*-High expression was associated with poor survival as measured by both Log Rank and Cox Proportional Hazard models in five cancer types. **f** Representative Kaplan–Meier survival curves for overall survival of BLCA, LIHC, LGG, LUSC, and UVM cancer types. **g** Forest plot of association between *NFATc2*-High or *NFATc2*-Low expressers and progression-free survival for 30 datasets with valid outcomes data. *NFATc2*-High expression was associated with poor progression-free survival as measured by both Log Rank and Cox Proportional Hazard models in three cancer types. **h** Representative Kaplan–Meier survival curves for progression-free survival of BLCA, LGG, and CESC cancer types. **i**) In the combined TCGA dataset as well as in both datasets demonstrating poor progression-free and overall survival in *NFATc2*-High expressers, *NFATc2*-High tumors were more likely to be considered High-grade. No difference was seen between *NFATc2*-High and *NFATc2*-Low expressers in terms of disease stage or metastatic status. ns not significant; *p ≤ 0.05; **p < 0.01; ***p < 0.001; ****p < 0.0001; *****p < 0.00001. ACC adrenocortical carcinoma, BLCA bladder urothelial carcinoma, BRCA breast invasive carcinoma, CESC cervical squamous cell carcinoma, CHOL cholangiocarcinoma, COAD colorectal adenocarcinoma, DLBC diffuse large B-cell lymphoma, ESCA esophageal adenocarcinoma, GBM glioblastoma multiforme, HNSC head and neck squamous cell carcinoma, KICH kidney chromophobe, KIRC kidney renal clear cell carcinoma, KIRP kidney renal papillary cell carcinoma, LAML acute myeloid leukemia, LGG brain lower grade glioma, LIHC liver hepatocellular carcinoma, LUAD lung adenocarcinoma, LUSC lung squamous cell carcinoma, MESO mesothelioma, OV ovarian serous cystadenocarcinoma, PAAD pancreatic adenocarcinoma, PCPG pheochromocytoma and paraganglioma, PRAD prostate adenocarcinoma, READ colorectal adenocarcinoma, SARC sarcoma, SKCM skin cutaneous melanoma, STAD stomach adenocarcinoma, TGCT testicular germ cell tumors, THCA thyroid carcinoma, THYM thymoma, UCEC uterine corpus endometrial carcinoma, UCS uterine carcinosarcoma, UVM uveal melanoma.

In 12 (36.4%) datasets, *RICTOR* was the top dysregulated upstream regulator as identified by IPA, demonstrating significant predicted signaling activation in all 12 cancer types. In total, 24 of the 33 TCGA datasets (72.7%) exhibited statistically significant activation of *RICTOR* signaling in *NFATc2*-High cancers (Fig. 4a). The magnitude of the difference in *NFATc2* expression between *NFATc2*-High and *NFATc2*-Low samples in each dataset was highly correlated with the estimated activation of *RICTOR* (Pearson coefficient = 0.61, *p* = 0.0002; Fig. 4b). In the Breast Invasive Carcinoma (BRCA) and Lung Adenocarcinoma (LUAD) datasets, selecting multiple percentile cutoffs (Supplementary Table 4) demonstrated the connection between the magnitude of the difference in *NFATc2* expression between *NFATc2*-High and *NFATc2*-Low samples, and the estimated activation of RICTOR (BRCA: Pearson coefficient = 0.91, *p* = 0.01; LUAD: Pearson coefficient = 0.91, *p* = 0.01; Fig. 4c). Additionally, 25 (75.5%) datasets exhibited significant upregulation of mTOR pathway (Fig. 4d). *RICTOR* was a major driver of mTOR pathway activation, with 21 (63.6%) datasets demonstrating both significantly activated mTOR and RICTOR (<0.0001). Taken together, *NFATc2* mRNA expression appears to have a biologic connection with the mTOR pathway across cancer types.

To assess for an association between *NFATc2* expression and clinical features across the TCGA datasets, high or low *NFATc2* mRNA expression was selected for each dataset in the top and bottom quartile of *NFATc2* expression (Supplementary Table 5). *NFATc2*-High tumors were statistically associated with poor overall survival in five datasets (bladder urothelial carcinoma [BLCA], liver hepatocellular carcinoma [LIHC], brain lower grade glioma [LGG], lung squamous cell carcinoma [LUSC], and uveal melanoma [UVM]; Fig. 4e, f) and poor progression-free survival (PFS) in three datasets (BLCA, LGG, and cervical squamous cell carcinoma [CESC]; Fig. 4g, h). A notable exception was higher *NFATc2* expression associated with improved PFS in the skin cutaneous melanoma (SKCM) dataset. In the combined TCGA dataset as well as in both datasets demonstrating poor progression-free and overall survival in *NFATc2*-High expressers, BLCA and LGG, *NFATc2*-High tumors were more likely to be considered high-grade (BLCA: 98.0% vs. 88.2%, *p* = 0.002; LGG: 64.3% vs. 45.7%, *p* = 0.003; All TCGA: 21.9% vs. 20.7%, *p* = 0.05; Fig. 4i). No difference was seen between *NFATc2*-High and *NFATc2*-Low expressers in terms of disease stage or metastatic status.

### Case report: mTOR inhibition-based therapy in an EWSR1-NFATc2 fusion positive sarcoma

**Fig. 5:**
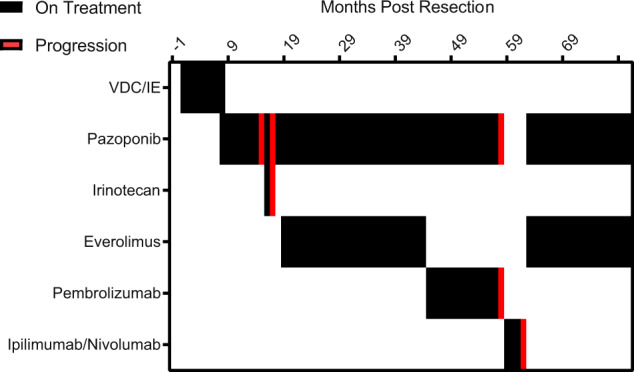
Treatment timeline of a 58-year-old man diagnosed with an *EWSR1-NFATc2* fusion positive sarcoma. Treatment history for a 58-year-old man diagnosed with an *EWSR1-NFATc2* gene fusion positive sarcoma. Genomic characterization also revealed a single pathogenic variant in *FANCE*. Briefly, following surgical excision, this patient received little benefit from adjuvant therapy of alternating vincristine, doxorubicin, and cyclophosphamide (VDC), followed by ifosfamide and etoposide (IE). Pazopanib alone and in combination with irinotecan provided little clinical benefit. Combination treatment with pazopanib and everolimus stabilized disease for 26 months before progression. Pembrolizumab in combination with pazopanib then stabilized disease for ten months before further disease progression, while further therapy with ipilimumab plus nivolumab provided disease stabilization for four months. The patient was then re-challenged with pazopanib and everolimus and has continued on this therapy for 21 months without progression before passing away due to causes unrelated to his active disease.

A 58-year-old man presented to the emergency room with hematochezia with subsequent anemia. Further workup revealed constriction of the transverse colon and invasion of the stomach by a 10-cm hypermetabolic mass. FDG-PET imaging showed regional hyper-metabolic nodules, which were of concern for advanced disease. The patient was diagnosed with a high-grade small round-cell tumor positive for CD99, CK AE1/3, vimentin, and Sox-9 on immunohistochemistry. Molecular analysis by fluorescent in situ hybridization revealed an *EWSR1* fusion. Comprehensive genomic profiling (CGP) identified an *EWSR1-NFATc2* fusion and revealed a single pathogenic variant in *FANCE* (Supplementary Table 6). Full treatment course for this patient is available in Fig. 5.

Briefly, initial therapy included surgical excision of the mass with residual disease necessitating adjuvant therapy of four cycles of alternating vincristine, dactinomycin, and cyclophosphamide, followed by ifosfamide and etoposide (VDC/IE). Adjuvant therapy provided stabilization of disease with some reduction of standard uptake values (SUV) by PET imaging but persistent low area of SUV as well as the presence of a hypermetabolic lesion concerning for progressive disease lead to initiation of pazopanib (400 mg once daily). Due to cumulative toxicities from adjuvant therapy and baseline cardiac dysfunction, traditional cytotoxic chemotherapy was not appropriate for further treatment. Four months later, PET scans revealed progression of disease with multiple new abdominal nodules. Combination treatment with pazopanib and irinotecan was initiated for 2 months but was not tolerated by the patient. Combination treatment with pazopanib and everolimus (5 mg once daily) was initiated based on data suggesting mTOR activation post-progression on VEGF-inhibiting therapy^36–39^. Pazopanib and everolimus caused a significant decrease in SUV in the target lesions by PET, but was matched with mixed response as measured by CT with an initial decrease in target lesion growth rate, before a steady increase in growth (Supplementary Fig. 4a, b). Treatment was complicated by multiple small bowl obstructions due to sarcomatosis resulting in prolonged breaks in therapy. Disease was considered stabilized as measured by PET scan on this combination for a total of 26 months before therapy was discontinued due to insurance issues. Also due to patient’s insurance, further PET scans to measure disease response were denied. Pembrolizumab (2 mg/kg) in combination with pazopanib was initiated with significant reduction in target lesions (Supplementary Fig. 4c) but were also noted to have development of additional lesions throughout the retroperitoneum and lung. Stabilization of overall disease for 10 months was followed by further disease progression, resulting in the need for a gastric tube. Salvage immunotherapy with ipilimumab (3 mg/kg) and nivolumab (1 mg/kg) was attempted for disease stabilization but the patient progressed on this regimen. The patient was then re-challenged with everolimus (5 mg daily) and pazopanib on a 400 mg week on-week off cycle. He had continued on this therapy for 21 months without an increase in size of any lesion or the development of any additional lesions before passing away from causes unrelated to his active disease. In total, the patient received 47 months of everolimus-based therapy.

## Discussion

The Ewing family of tumors has previously been considered to include a number of cancers classified by similarity in recurrent oncogenic driver fusions^40,41^. Previous studies have served to demonstrate the molecular distinction between *BCOR-CCNB3*, *CIC-DUX4*, *EWSR1-PATZ1*, and *EWSR1-NFATc2* fusion positive sarcomas and other traditional Ewing tumors^7,26–28,30,42,43^. To date, this molecular distinction has not been assessed on the genomic level. Here, data from 1024 *EWSR1* fusion positive sarcomas demonstrates the uniqueness of *EWSR1-NFATc2* fusion cancers from other cancers in the Ewing family. In comparison to *EWSR1-FLI1 and EWSR1-ERG*, the secondary genomic landscape of *EWSR1-NFATc2* fusion positive sarcomas is significantly different and appears to be driven by genes related to the mTOR pathway. Reanalysis of data from two independent datasets including *CIC-DUX4* and *EWSR1-NFATc2 (*GSE60740^27^), and *EWSR1-ETS* (GSE34620^44^) positive tumors identified activation of the mTOR pathway in *EWSR1-NFATc2*, but not *CIC-DUX4* or *EWSR1-ETS* positive tumors. The original report comparing *CIC-DUX4* and *EWSR1-NFATc2* positive tumors identified key molecular markers differentiating the two diseases; presenting PAX7 as a highly specific marker for *EWSR1-NFATc2* positive tumor diagnosis. While this data serves to bolster previous findings of the unique nature of the *EWSR1-NFATc2* fusion, this study also associates *EWSR1-NFATc2* fusion positive sarcomas with activation of the mTOR pathway.

At the molecular level, the NFATc2 protein has been shown to directly associate with and regulate the mTOR pathway through *PTEN* and the *TORC1/2* complex^45–49^. Furthermore, RAPTOR, itself a regulator of mTOR activity, has been demonstrated to have a protein level interaction with NFATc2^46^. This provides a potential link between T-cell activation and proliferation in normal tissue; however, the link between these two pathways has not been well studied in the case on *NFATc2* fusions. Given the suggested activation of mTOR in *EWSR1-NFATc2* fusion positive sarcomas, and the conservation of the primary transactivation and regulatory domains of *NFATc2* in *EWSR1-NFATc2* fusion, we proceeded to assess the role of *NFATc2* overexpression on mTOR in a tumor agnostic fashion. Pan-cancer analyses utilizing large in vitro and clinical datasets suggest a strong correlation between *NFATc2* expression and mTOR pathway activation. Clinical data also suggest that *NFATc2* expression may correlate with tumor grade, consistent with previous reports noting increased tumor grade in mTOR-activated tumors^50,51^.

Furthermore, we report a clinical case of a patient with an *EWSR1-NFATc2* fusion positive sarcoma whose tumor was stabilized by mTOR combination therapy. This durable benefit is significant given the lack or reported efficacy of chemotherapeutic agents in advanced or metastatic disease of this type. This finding is limited due to the extent of the spread of the disease; with extensive sarcomatosis disrupting traditional measures of progression by RECIST criteria. Disease was primarily tracked by PET as the patient’s chronic renal insufficiency did not allow for CT with contrast. Accurately assessing the extent of disease was difficult and exact measurements were not always possible. Furthermore, after nearly 4 years post-diagnosis, the patient’s insurance denied further coverage of PET scans. While it is difficult to ascertain clinical stability due to indolent disease versus effective therapy, progression of disease off therapy suggests the combination of everolimus and pazopanib had an effect on this patient’s tumor. While this finding is not suggestive of exquisite stabilization of *EWSR1-NFATc2* fusion positive sarcomas to mTOR combination therapy, when linked with the other presented evidence a case could be made for mTOR as a potential therapeutic target in this disease.

This report represents preliminary evidence to support potential directions for targeted therapeutic study in *EWSR1-NFATc2* fusion positive sarcomas where formal evaluation of mTOR-directed therapies have not been performed. This study is limited by the inherent nature of retrospective studies. Small datasets of this rare fusion sarcoma further reduce the external validity of the finding of this study. No current cell lines exist to validate these findings in vitro. Further research is necessary to provide conclusive recommendations for the clinical treatment of patients battling this rare sarcoma.

In this study, the largest multi-omic assessment of *EWSR1-NFATc2* fusion positive sarcomas to date, our data reinforces previous findings that *EWSR1-NFATc2* fusion positive sarcomas are molecularly distinct from standard Ewing sarcomas. Genomic and transcriptomic level data pinpoint key dysregulation in the mTOR pathway that may be therapeutically viable. Prospective clinical evaluation will be required to validate these findings. Taken together, these findings support the potential clinical application of precision medicine in the Ewing family of tumors.

## Methods

### Comprehensive genomic profiling data

CGP data from *EWSR1* fusion positive subjects, whose tumors were assayed in the course of clinical care using FMI hybrid-capture-based next-generation sequencing platform, was provided as previously described^52–54^. Approval for the retrospective collection of genomic data from FMI, including a waiver of informed consent and a HIPAA waiver of authorization, was obtained from the Western Institutional Review Board (protocol no. 20152817).

*EWSR1* fusions and individual partner genes were identified by RNA sequencing. From this database, microsatellite instability (MSI), tumor mutation burden (TMB), and pathogenicity of genomic variants were determined utilizing FMI’s analysis pipeline. Variants referred to as known or likely pathogenic (pathogenic variants), and variants of unknown significance (VUS) were included in this analysis. Full mRNA sequences for the *EWSR1*-*NFATc2* fusion were provided for analysis of fusion sites as well as conserved regions of the *NFATc2* gene. Analysis of genomic similarity between *EWSR1* fusions samples was conducted by finding the frequency of gene alterations by fusion partner gene.

### Gene expression in EWSR1-NFATc2 and EWSR1-ETS positive tumors

Data from two independent datasets assessing baseline gene expression in *CIC-DUX4* and *EWSR1-NFATc2* (GSE60740^27^; *n* = 14 and *n* = 7), and *EWSR1-ETS* (GSE34620^44^; *n* = 117) positive tumors were obtained from the Gene Expression Omnibus (https://www.ncbi.nlm.nih.gov/geo, downloaded April 10, 2019). *CIC-DUX4* fusion positive tumors where once thought to be part of the Ewing family of tumors; however, these tumors have been demonstrated to be unique entities. The *CIC-DUX4* fusion positive samples for GSE60740 were included as controls in the comparison between *EWSR1-NFATc2* and *EWSR1-ETS* positive tumors. Both studies used the Affymetrix U133A microarray. Datasets were combined before being processed in R using robust multichip average (RMA) normalization. Differential expression between each combination of the three fusion sets (*CIC-DUX4* vs. *EWSR1-NFATc2*; *EWSR1-NFATc2* vs. *EWSR1-ETS*; *CIC-DUX4* vs. *EWSR1-ETS*) was followed by pathway analysis.

### Genomics of drug sensitivity in cancer database

Data, including DNA variants, mRNA expression, and drug sensitivity, was collected from the genomics of drug sensitivity in cancer (http://cancerrxgene.org, downloaded August 22, 2019)^55,56^. Cell lines identified to have known cancer driver genomic variants in *PTEN*, *mTOR*, *TSC1*, or *TSC2* were excluded from our analysis to reduce known covariates to mTOR pathway activity. Cell lines were divided into *NFATc2*-Low and *NFATc2*-High categories by median *NFATc2* expression. Differential expression was followed by pathway analysis.

### The cancer genome atlas

DNA copy number, mRNA expression, and clinical data from 33 non-overlapping TCGA datasets were obtained from firebrowse.org (downloaded October 28, 2019)^57,58^. To assess for the effect of extreme *NFATc2* expression, high and low *NFATc2* expressing samples were selected by identifying the fewest number of samples (highest and lowest) necessary to identify a statistically significant difference between high and low samples, (adjusted *p* value < 0.05) with no less than ten samples in each of the *NFATc2-*High and *NFATc2-*Low cohorts. Once the percentile cutoffs were identified for each dataset, differential expression and pathway analysis were conducted.

### Statistical methods

All data was analyzed in R_v.3.4.3_ or Graphpad Prism_v.8.0.0_. Two sided Student’s *t*-test, one-way ANOVA, and Chi-squared tests were used as appropriate. Continuous data are presented as mean ± SEM unless otherwise stated. To test the correlation between rapamycin sensitivity in *NFATc2-*High and *NFATc2-*Low cell lines from the genomics of drug sensitivity in cancer, we used an unpaired *t* test with Welch’s correction both for it appropriateness to the data as well as its conservative results. Survival analysis was tested using both log-rank and Cox proportional hazard methods. Survival graphs were created using the Kaplan–Meier estimator. Principle component analysis (PCA) was conducted for gene variant data using the prevalence of known pathogenic variants for each *EWSR1* fusion partner as variable values using the FactoMineR_v2.3_ package in R^59^. Differential expression was conducted in R with the limma_v3.40.6_ package^60^. Pathway analysis was conducted using ingenuity pathway analysis (IPA; Qiagen https://www.qiagenbioinformatics.com/products/ingenuitypathway-analysis) using genes identified by differential expression to have a log_2_ fold change of >1 or <−1 and an unadjusted *p*-value of <0.05 for each individual set^61^. IPA core analysis was executed using expression log ratio. IPA results were filtered to only include results for the mTOR pathway; therefore, unadjusted *p*-values were used to identify statistical significance. Ingenuity upstream regulator analysis in IPA was then used to identify the probable cascade of upstream transcriptional regulators that can explain the observed gene expression changes. Regulators for each dataset was sorted by *p*-value and activation *z*-score. Unless otherwise stated, *p*-values ≤0.05 were considered statistically significant. FDR was used as appropriate.

### Case report

A patient with a confirmed *EWSR1-NFATc2* fusion positive sarcoma provided written consent for the disclosure of this case report. Data was collected by chart review by two independent authors to ensure accuracy of data collection. A specialized sarcoma radiologist reviewed all available CT and CT/PET scans to quantify disease and assisted in the interpretation of the data. Genomic sequencing was conducted by FMI from a clinically obtained tumor biopsy. All data regarding this case report was generated during routine clinical care. No data was generated explicitly for the purpose of publication. This case report was approved by the Ohio State University IRB (IRB approval: 2014C0181).

### Reporting summary

Further information on research design is available in the Nature Research Reporting Summary linked to this article.

## Supplementary information

Reporting Summary

Supplementary Information

## Data Availability

The data generated and analyzed during this study are described in the following data record: 10.6084/m9.figshare.14270366^62^. This data record also contains a data file showing the genomic profiling of pathogenic variants in EWSR1-NFATc2 fusion positive sarcomas^62^. The gene expression data used in this study are openly available from the Gene Expression Omnibus repository^63,64^. The drug sensitivity data are openly accessible from the Genomics of Drug Sensitivity in Cancer Database (https://www.cancerrxgene.org/). The TCGA data are openly accessible from firebrowse.org (http://gdac.broadinstitute.org/runs/stddata__2016_01_28/data/), and the copy number data are accessible from cBioPortal (https://www.cbioportal.org/). Clinical and genomic case report data cannot be openly shared in order to protect patient confidentiality, but can be made available on request from James L. Chen (James.Chen@osumc.edu).
