# Supplementary material for: Multiscale-omic assessment of EWSR1-NFATc2 fusion positive sarcomas identifies the mTOR pathway as a potential therapeutic target
Source: NPJ Precis Oncol. 2021 May 21;5:43. doi: 10.1038/s41698-021-00177-0 (PMC8140100; doi:10.1038/s41698-021-00177-0)
Supplement: Supplementary file 1 — Reporting Summary [file 41698_2021_177_MOESM1_ESM.pdf]

nature research

Corresponding author(s): DBPR NPJPRECISIONONCOLOGY-00751

Last updated by author(s): Sep 8, 2020

Reporting Summary

Nature Research wishes to improve the reproducibility of the work that we publish. This form provides structure for consistency and transparency in reporting. For further information on Nature Research policies, see our Editorial Policies and the Editorial Policy Checklist.

Statistics

For all statistical analyses, confirm that the following items are present in the figure legend, table legend, main text, or Methods section.

n/a	Confirmed	
☐	☒	The exact sample size (*n*) for each experimental group/condition, given as a discrete number and unit of measurement
☐	☒	A statement on whether measurements were taken from distinct samples or whether the same sample was measured repeatedly
☐	☒	The statistical test(s) used AND whether they are one- or two-sided<br>*Only common tests should be described solely by name; describe more complex techniques in the Methods section.*
☐	☒	A description of all covariates tested
☐	☒	A description of any assumptions or corrections, such as tests of normality and adjustment for multiple comparisons
☐	☒	A full description of the statistical parameters including central tendency (e.g. means) or other basic estimates (e.g. regression coefficient) AND variation (e.g. standard deviation) or associated estimates of uncertainty (e.g. confidence intervals)
☐	☒	For null hypothesis testing, the test statistic (e.g. *F*, *t*, *r*) with confidence intervals, effect sizes, degrees of freedom and *P* value noted<br>*Give P values as exact values whenever suitable.*
☒	☐	For Bayesian analysis, information on the choice of priors and Markov chain Monte Carlo settings
☒	☐	For hierarchical and complex designs, identification of the appropriate level for tests and full reporting of outcomes
☒	☐	Estimates of effect sizes (e.g. Cohen's *d*, Pearson's *r*), indicating how they were calculated

Our web collection on statistics for biologists contains articles on many of the points above.

Software and code

Policy information about availability of computer code

Data collection	No software was used to collect data.
Data analysis	All data was analyzed in Rv.3.4.3 or Graphpad Prismv.8.0.0.

For manuscripts utilizing custom algorithms or software that are central to the research but not yet described in published literature, software must be made available to editors and reviewers. We strongly encourage code deposition in a community repository (e.g. GitHub). See the Nature Research guidelines for submitting code & software for further information.

Data

Policy information about availability of data

All manuscripts must include a data availability statement. This statement should provide the following information, where applicable:

- Accession codes, unique identifiers, or web links for publicly available datasets
- A list of figures that have associated raw data
- A description of any restrictions on data availability

The data generated and analyzed during this study are described in the following data record: https://doi.org/10.6084/m9.figshare.14270366. This data record also contains a data file showing the genomic profiling of pathogenic variants in EWSR1-NFATc2 fusion positive sarcomas. The gene expression data used in this study are openly available from the Gene Expression Omnibus repository. The drug sensitivity data are openly accessible from the Genomics of Drug Sensitivity in Cancer Database (https://www.cancerrxgene.org/). The TCGA data are openly accessible from firebrowse.org (http://gdac.broadinstitute.org/runs/stddata__2016_01_28/data/), and the copy number data are accessible from cBioPortal (https://www.cbioportal.org/). Clinical and genomic case report data cannot be openly shared in order to protect patient confidentiality, but can be made available on request from James L. Chen (James.Chen@osumc.edu).

Field-specific reporting

Please select the one below that is the best fit for your research. If you are not sure, read the appropriate sections before making your selection.

- [x] Life sciences
- [ ] Behavioural & social sciences
- [ ] Ecological, evolutionary & environmental sciences

For a reference copy of the document with all sections, see nature.com/documents/nr-reporting-summary-flat.pdf

Life sciences study design

All studies must disclose on these points even when the disclosure is negative.

Sample size	Sample size was not determined in this study. All data available were used for the analysis presented here.
Data exclusions	No data was excluded for this study.
Replication	No designated replication studies were conducted; however, analysis from multiple data sources were used to validate the collective findings of this study.
Randomization	N/A
Blinding	N/A

Reporting for specific materials, systems and methods

We require information from authors about some types of materials, experimental systems and methods used in many studies. Here, indicate whether each material, system or method listed is relevant to your study. If you are not sure if a list item applies to your research, read the appropriate section before selecting a response.

Materials & experimental systems

n/a	Involved in the study	
☒	☐	Antibodies
☒	☐	Eukaryotic cell lines
☒	☐	Palaeontology and archaeology
☒	☐	Animals and other organisms
☒	☐	Human research participants
☐	☒	Clinical data
☒	☐	Dual use research of concern

Methods

n/a	Involved in the study	
☒	☐	ChIP-seq
☒	☐	Flow cytometry
☒	☐	MRI-based neuroimaging

Clinical data

Policy information about clinical studies

All manuscripts should comply with the ICMJE guidelines for publication of clinical research and a completed CONSORT checklist must be included with all submissions.

Clinical trial registration	N/A
Study protocol	N/A
Data collection	Data was collected retrospectively for the clinical course of a single patient reported here as a case report.
Outcomes	Pre-defined clinical outcomes for the case reported here were selected to clearly demonstrate the specific clinical course of the patient.
