# Supplementary material for: Multiscale-omic assessment of EWSR1-NFATc2 fusion positive sarcomas identifies the mTOR pathway as a potential therapeutic target
Source: NPJ Precis Oncol. 2021 May 21;5:43. doi: 10.1038/s41698-021-00177-0 (PMC8140100; doi:10.1038/s41698-021-00177-0)
Supplement: Supplementary file 2 — Supplementary Information [file 41698_2021_177_MOESM2_ESM.pdf]

Supplemental Materials

Supplementary Table 1: Summary of Treatment of Known Cases of EWSR1-NFATc2 Fusion Positive Sarcoma

Supplementary Table 2: Secondary Genomic Variants in EWRS1-NFATc2 Fusion Positive Samples

Supplementary Figure 1: Gene Contribution of Principle Component Analysis

Supplementary Figure 2: Gene expression suggests unique biology of EWSR1-NFATc2 positive sarcomas

Supplementary Figure 3: NFATc2 genomic amplification in 33 non-overlapping TCGA datasets

Supplementary Table 3: TCGA Dataset Cutoffs

Supplementary Table 4: BRCA and LUAD Multiple Percentile Cutoffs

Supplementary Table 5: TCGA Survival Analysis

Supplementary Table 6: Genomic Test Result

Supplementary Figure 4: PET Scans – Case Report

Supplementary Table 1: Summary of Treatment of Known Cases of EWSR1-NFATc2 Fusion Positive Sarcoma				
	Age at Diagnosis (Years)	Sex	Site	Treatment Summary
Case #1[6]	24	Female	Soft-tissue - Lower Extremity	Neoadjuvant chemotherapy consisting of five cycles of alternating doxorubicin/vincristine and cyclophosphamide/ifosfamide/etoposide with no significant response followed by resection. At 12 month follow-up no disease was appreciable.
Case #2[20]	12	Male	Bone - Femur	Neoadjuvant chemotherapy stated to be clinically ineffective followed by resection with total femoral prosthesis. 11 months follow-up no evidence of disease
Case #3[20]	28	Male	Bone - Humerus	Neoadjuvant Chemotherapy followed by resection with bone graft; Local recurrence 4 years later with suspected lung metastasis, Alive With Disease at 5-mo follow-up
Case #4[21]	32	Male	Lower extremity	Radiotherapy for suspected N-HL of bone 7 years prior to initial diagnosis; no evidence of disease at 64-mo follow-up
Case #5[18]	30	Male	Bone - Femur	Initial treatment by curettage of bone lesion followed by a bone graft resulted in 2.5 years of disease control. Recurrence was treated by resection followed by adjuvant chemotherapy with etoposide/ifosfamide for 3 months. Four months following adjuvant therapy scans revealed no evidence of disease.
Case #6[7]	43	Male	Bone - Femur	Initial diagnosis of “necrotic tumor suggestive of plasmacytoma” was treated by radical radiotherapy resulting in no response. Re-diagnosis of Ewing’s Sarcoma was then treated with neoadjuvant chemotherapy consisting of ifosfamide/vincristine/dactinomycin/doxorubicin with minimal response, resection, and adjuvant chemotherapy with methotrexate. At 12 month follow-up no disease was appreciable.

Supplementary Table 2: Secondary Genomic Variants in EWRS1-NFATc2 Fusion Positive Samples			
**Gene**	**Variant**	**Functional Status**	**Functional Call**
ACTB	P332S	Unknown	Variant of unknown significance
AKT3	Amplification	Known	Known pathogenic variant
ALOX12B	I685V	Unknown	Variant of unknown significance
APCDD1	Amplification	Unknown	Variant of unknown significance
ASXL1	Amplification	Unknown	Variant of unknown significance
ATR	splice	Likely	Known pathogenic variant
BCL10	A216T	Unknown	Variant of unknown significance
BCL2L1	Amplification	Known	Known pathogenic variant
BCOR	T913fs*8	Likely	Known pathogenic variant
BCOR	A220S	Unknown	Variant of unknown significance
CD22	R511Q	Known	Known pathogenic variant
CDKN2A	Deletion	Known	Known pathogenic variant
CDKN2A - CEP55	Truncation	Likely	Known pathogenic variant
CDKN2B	Deletion	Known	Known pathogenic variant
CEBPA	A25V	Unknown	Variant of unknown significance
CHEK2	Amplification	Unknown	Variant of unknown significance
CHUK	R219S	Unknown	Variant of unknown significance
EED	E90*	Likely	Known pathogenic variant
EP300	R649K	Unknown	Variant of unknown significance
EPHA3	Amplification	Known	Known pathogenic variant
EPHA5	Amplification	Unknown	Variant of unknown significance
ERBB4	T926M	Known	Known pathogenic variant
FAF1	Q277R	Unknown	Variant of unknown significance
FANCD2	K811N	Unknown	Variant of unknown significance
FANCE	V311fs*2	Likely	Known pathogenic variant
FANCG	G276R	Known	Known pathogenic variant
FBXO11	Q56_P57insQ	Unknown	Variant of unknown significance
FBXO11	Y142C	Unknown	Variant of unknown significance
FLT4	A360V	Unknown	Variant of unknown significance
FRS2	Amplification	Known	Known pathogenic variant
HIST1H1E	A123G	Unknown	Variant of unknown significance
HSP90AA1	K369_E370del	Unknown	Variant of unknown significance
INHBA	K272N	Unknown	Variant of unknown significance
INPP4B	I400V	Unknown	Variant of unknown significance
INPP5D	S713F	Unknown	Variant of unknown significance
IRS2	R137S	Unknown	Variant of unknown significance
KAT6A	P1277L	Unknown	Variant of unknown significance
KAT6A	P970S	Unknown	Variant of unknown significance
KDM5A	R145H	Unknown	Variant of unknown significance

KDR	Amplification	Known	Known pathogenic variant
KDR	Amplification	Ambiguous	Variant of unknown significance
KDR	N534fs*9	Unknown	Variant of unknown significance
KIT	Amplification	Known	Known pathogenic variant
KIT	Amplification	Ambiguous	Variant of unknown significance
KLHL6	M123I	Unknown	Variant of unknown significance
KMT2A	A2968fs*1	Unknown	Variant of unknown significance
KMT2C	H322D	Unknown	Variant of unknown significance
KMT2C	I830V	Unknown	Variant of unknown significance
LRP1B	D3105H	Unknown	Variant of unknown significance
MAGED1	A822T	Unknown	Variant of unknown significance
MDM2	Amplification	Known	Known pathogenic variant
MSH3	A60_A62del	Unknown	Variant of unknown significance
MTOR	E1799K	Known	Known pathogenic variant
MTOR	R1161Q	Known	Known pathogenic variant
NCOR2	V37I	Unknown	Variant of unknown significance
NF1	R1968*	Known	Known pathogenic variant
NF1	T586fs*18	Likely	Known pathogenic variant
PARP2	D32fs*10	Unknown	Variant of unknown significance
PASK	E651*	Likely	Known pathogenic variant
PCLO	P1895S	Known	Known pathogenic variant
PDCD11	E1449del	Unknown	Variant of unknown significance
PDGFRA	Amplification	Known	Known pathogenic variant
PDGFRA	Amplification	Ambiguous	Variant of unknown significance
RELN	S1014G	Unknown	Variant of unknown significance
RELN	S1673R	Unknown	Variant of unknown significance
RICTOR	Amplification	Known	Known pathogenic variant
RMRP	Rearrangement	Unknown	Variant of unknown significance
ROS1	K146T	Unknown	Variant of unknown significance
SDHD	Q47R	Unknown	Variant of unknown significance
SF3B1	V55M	Unknown	Variant of unknown significance
SMARCB1	Amplification	Unknown	Variant of unknown significance
SMARCD1	Amplification	Unknown	Variant of unknown significance
SMC3	S611N	Unknown	Variant of unknown significance
SRC	Amplification	Known	Known pathogenic variant
TCF3	R487Q	Unknown	Variant of unknown significance
TOP1	Amplification	Known	Known pathogenic variant
TP53	E294*	Known	Known pathogenic variant
TP53	R282W	Known	Known pathogenic variant
TP53	Truncation	Likely	Known pathogenic variant
TRAF3	V96I	Unknown	Variant of unknown significance
TSHR	G21R	Unknown	Variant of unknown significance
VHL	R200W	Known	Known pathogenic variant

XBP1	Amplification	Unknown	Variant of unknown significance

Supplementary Figure 1: Gene Contribution of Principle Component Analysis

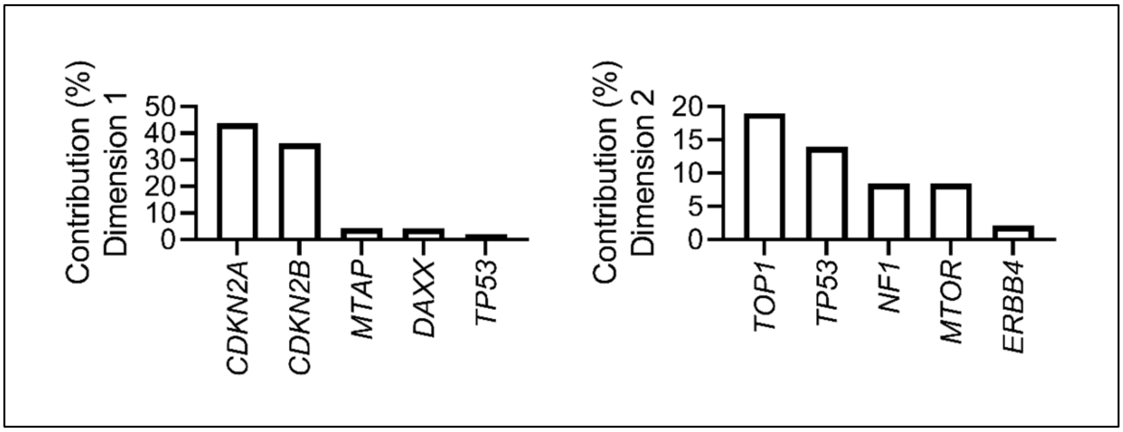

Principle component analysis identified dis-similarity between secondary genomic landscapes of *EWSR1* fusion positive sarcomas. Contribution to each of the first two dimensions of the PCA analysis are presented here.

Supplementary Figure 2: Gene expression suggests unique biology of *EWSR1-NFATc2* positive sarcomas

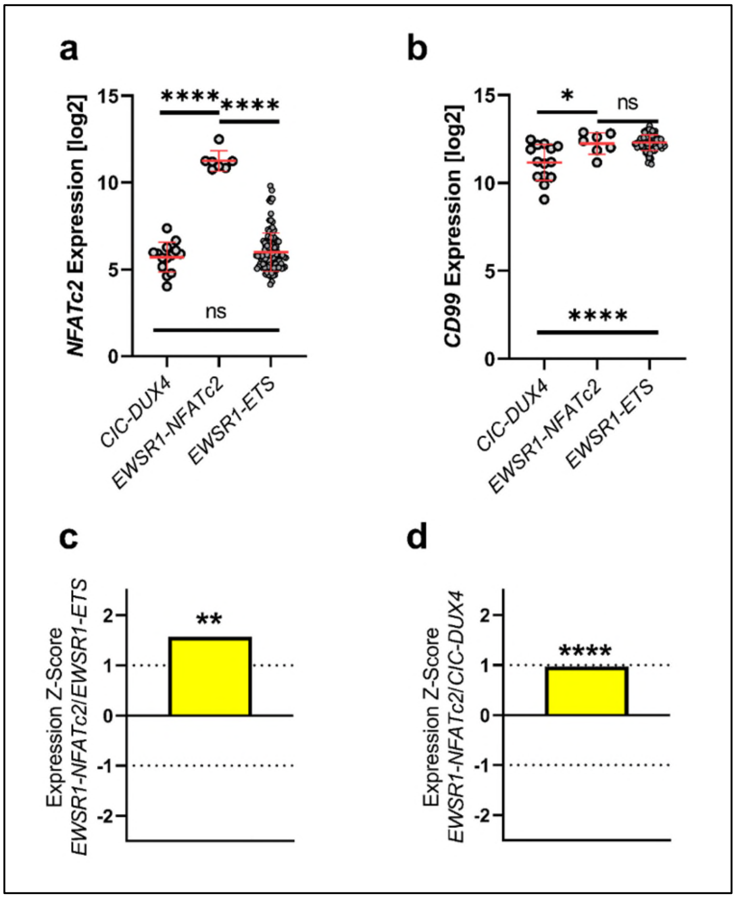

Data from 7 *EWSR1-NFATc2* positive sarcomas, 14 *CIC-DUX4* positive sarcomas, and *117 EWSR1-ETS* positive sarcomas downloaded from the GEO database. **a**) NFATc2 was significantly overexpressed in *EWSR1-NFATc2* positive sarcomas (Mean±SD: *EWSR1-NFATc2* 11.3±0.6, *CIC-DUX4* 5.7±0.9, *EWSR1-ETS* 5.9±1.1, *EWSR1-NFATc2* vs *EWSR1-ETS* p<0.0001, *EWSR1-NFATc2* vs *CIC-DUX4* p<0.0001, *CIC-DUX4* vs *EWSR1-ETS* p=0.34). **b**) *CD99*, a cell surface marker associated with Ewing sarcoma, was equally expressed in *EWSR1-NFATc2* compared to *EWSR1-ETS* positive sarcomas, but lowly expressed in *CIC-DUX4* positive sarcomas (Mean±SD: *EWSR1-NFATc2* 12.3±0.6, *CIC-DUX4* 11.2±1.0, *EWSR1-ETS* 12.3±0.4, *EWSR1-NFATc2* vs *EWSR1-ETS* p=0.81, *EWSR1-NFATc2* vs *CIC-DUX4* p= 0.02, *CIC-DUX4* vs *EWSR1-ETS* p <0.0001). **c and d**) Pathway analysis identified a significant activation of the mTOR pathway in *EWSR1-NFATc2* positive sarcomas compared to either *EWSR1-ETS* (*EWSR1-NFATc2* vs *EWSR1-ETS*: z-score=1.6, p=0.002, **c**), or *CIC-DUX4* positive sarcomas (*EWSR1-NFATc2* vs *CIC-DUX4*: z-score=1, p<0.001; **d**). No discernable difference in mTOR pathway activation was identified between *EWSR1-ETS* and *CIC-DUX4* positive sarcomas (*EWSR1-ETS* vs *CIC-DUX4*: z-score= -0.2, p=0.01).

ns: Not Significant; * p ≤ 0.05; ** p < 0.01; *** p < 0.001; **** p <0.0001; ***** p <0.00001

Supplementary Figure 3: *NFATc2* genomic amplification in 33 non-overlapping TCGA datasets

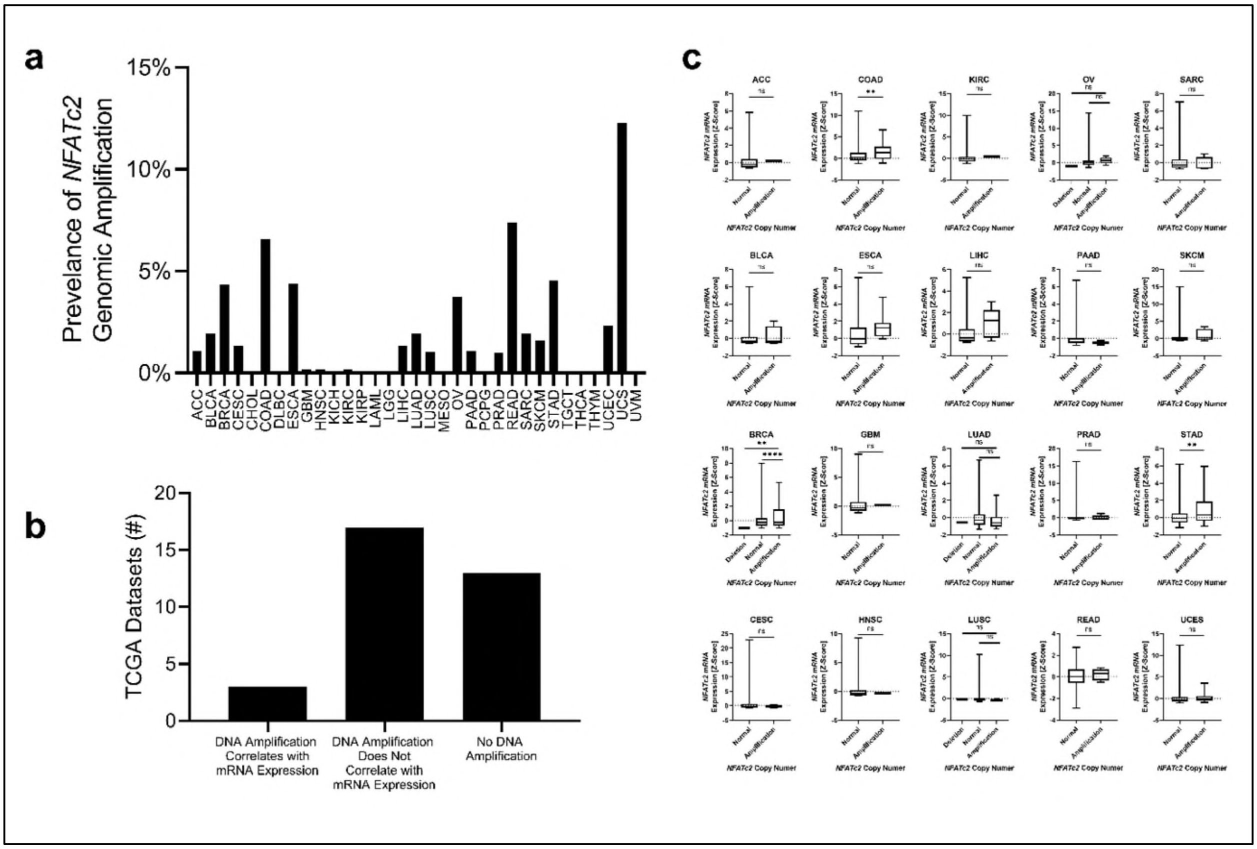

a) Prevalence of *NFATc2* genomic amplification in 33 non-overlapping TCGA datasets. **b-c**) Pan-cancer TCGA assessment suggests poor correlation between *NFATc2* genomic amplification status and *NFATc2* mRNA expression (**b**). Representative plots of *NFATc2* mRNA expression by *NFATc2* genomic amplification status for datasets with samples positive for an *NFATc2* genomic amplification (**c**).

ns: Not Significant; * $p \leq 0.05$; ** $p < 0.01$; *** $p < 0.001$; **** $p < 0.0001$; ***** $p < 0.00001$

Supplementary Table 3: TCGA Dataset Cutoffs							
Dataset	Percentile Cutoff	*NFATc2*-High		*NFATc2*-Low		Fold Change	Adjusted p Value
		n	Mean *NFATc2* Expression	n	Mean *NFATc2* Expression		
ACC	12%	10	42.54	10	0.05	885.6	1.26E-03
BLCA	4%	18	220.07	18	0.07	3367.9	1.85E-07
BRCA	1%	13	638.02	13	0.20	3251.6	7.33E-07
CESC	6%	19	157.81	19	0.07	2194.2	3.88E-03
CHOL	22%	10	153.11	10	7.91	19.4	9.88E-03
COAD	2%	10	373.01	10	2.05	181.7	1.84E-06
DLBC	20%	10	542.54	10	6.60	82.2	8.01E-06
ESCA	5%	10	980.40	10	9.64	101.7	1.21E-05
GBM	6%	10	171.70	10	2.66	64.7	8.85E-04
HNSC	3%	17	419.04	17	0.11	3958.0	1.17E-07
KICH	11%	10	221.84	10	5.05	43.9	4.95E-03
KIRC	6%	37	486.42	37	7.25	67.1	1.84E-02
KIRP	5%	17	179.09	17	0.10	1811.7	1.66E-09
LAML	6%	10	1208.79	10	49.63	24.4	2.03E-07
LGG	2%	10	321.52	10	0.26	1255.6	3.04E-04
LIHC	8%	34	186.91	34	0.08	2407.9	3.46E-20
LUAD	2%	12	449.06	12	2.23	201.4	9.39E-04
LUSC	2%	12	522.50	12	0.68	766.6	1.10E-03
MESO	13%	12	381.12	12	2.19	174.2	3.77E-02
OV	3%	10	584.96	10	17.18	34.0	3.12E-02
PAAD	5%	10	621.45	10	4.41	141.0	4.16E-03
PCPG	5%	10	223.04	10	0.18	1212.7	1.76E-03
PRAD	2%	11	233.14	11	0.08	3036.7	3.73E-08
READ	9%	10	281.15	10	9.55	29.4	2.28E-04
SARC	4%	10	288.77	10	0.24	1200.9	5.43E-05
SKCM	2%	10	2402.06	10	0.97	2469.8	1.25E-03
STAD	2%	10	1681.65	10	32.62	51.5	3.09E-06
TGCT	6%	10	197.42	10	1.02	193.1	1.14E-03
THCA	2%	12	555.47	12	0.78	711.2	1.47E-02
THYM	8%	10	409.91	10	0.48	856.4	1.86E-03
UCEC	5%	10	131.13	10	0.07	1898.5	8.47E-06
UCS	22%	13	58.14	13	1.12	52.0	4.30E-02
UVM	12%	10	36.70	10	0.33	112.3	1.23E-02

Supplementary Table 4: BRCA and LUAD Multiple Percentile Cutoffs							
Dataset	Percentile Cutoff	*NFATc2*-High		*NFATc2*-Low		Fold Change	p Value
		n	Mean *NFATc2* Expression	n	Mean *NFATc2* Expression		
BRCA	1%	13	638.02	13	0.20	3251.6	1.47E-08
	2%	25	511.12	25	0.62	822.1	5.51E-13
	5%	61	375.78	61	1.65	227.6	9.36E-26
	10%	122	294.40	122	3.89	75.8	2.72E-45
	25%	303	204.99	303	13.71	14.9	6E-88
	50%	606	147.96	606	31.16	4.7	1.1E-113
LUAD	1%	6	575.17	6	0.78	741.2	0.003
	2%	12	449.06	12	2.23	201.4	1.88E-05
	5%	29	335.44	29	5.05	66.4	2.48E-11
	10%	58	273.02	58	8.95	30.5	1.55E-21
	25%	144	202.76	144	20.29	10.0	2.86E-46
	50%	288	151.73	288	39.23	3.9	3.96E-61

Supplementary Table 5: TCGA Survival Analysis						
Dataset	Progression-Free Survival			Overall Survival		
	Hazard-Ratio (HR) and 95% Confidence Interval (95%CI)	p value		Hazard-Ratio (HR) and 95% Confidence Interval (95%CI)	p value	
		Log-rank	Cox Proportional Hazard		Log-rank	Cox Proportional Hazard
ACC	HR 1.05 [95%CI 0.47 - 2.33]	0.89	0.89	HR 1.02 [95%CI 0.36 - 2.84]	0.96	0.96
BLCA	HR 1.53 [95%CI 0.99 - 2.35]	0.05	0.05	HR 1.49 [95%CI 0.98 - 2.27]	0.05	0.05
BRCA	HR 0.71 [95%CI 0.45 - 1.09]	0.12	0.12	HR 1.37 [95%CI 0.88 - 2.13]	0.15	0.15
CESC	HR 2.18 [95%CI 1.02 - 4.64]	0.03	0.04	HR 1.84 [95%CI 0.87 - 3.89]	0.1	0.1
CHOL	HR 0.62 [95%CI 0.16 - 2.40]	0.48	0.49	HR 0.67 [95%CI 0.15 - 3.05]	0.61	0.61
COAD	HR 1.49 [95%CI 0.74 - 3.03]	0.25	0.26	HR 1.39 [95%CI 0.63 - 3.04]	0.41	0.4
DLBC	HR 1.92 [95%CI 0.34 - 10.6]	0.43	0.45	HR 0.90 [95%CI 0.05 - 14.5]	0.94	0.94
ESCA	HR 0.76 [95%CI 0.38 - 1.52]	0.45	0.45	HR 1.12 [95%CI 0.55 - 2.30]	0.74	0.74
GBM	HR 1.43 [95%CI 0.87 - 2.34]	0.14	0.15	HR 1.21 [95%CI 0.74 - 1.98]	0.42	0.42
HNSC	HR 0.69 [95%CI 0.47 - 1.03]	0.07	0.07	HR 0.88 [95%CI 0.59 - 1.30]	0.52	0.52
KICH	HR 1.43 [95%CI 0.31 - 6.43]	0.63	0.64	HR 0.94 [95%CI 0.13 - 6.72]	0.95	0.95
KIRC	HR 0.94 [95%CI 0.59 - 1.51]	0.82	0.82	HR 0.70 [95%CI 0.45 - 1.09]	0.12	0.12
KIRP	HR 1.23 [95%CI 0.57 - 2.62]	0.58	0.58	HR 1.31 [95%CI 0.50 - 3.41]	0.57	0.57
LAML	N/A			HR 1.04 [95%CI 0.59 - 1.83]	0.87	0.87
LGG	HR 2.06 [95%CI 1.35 - 3.12]	0.0004	0.0006	HR 2.53 [95%CI 1.45 - 4.41]	0.0005	0.001
LIHC	HR 1.25 [95%CI 0.82 - 1.90]	0.29	0.29	HR 1.76 [95%CI 1.05 - 2.96]	0.02	0.02
LUAD	HR 0.81 [95%CI 0.52 - 1.24]	0.33	0.34	HR 0.65 [95%CI 0.41 - 1.02]	0.05	0.06
LUSC	HR 1.41 [95%CI 0.89 - 2.25]	0.13	0.13	HR 1.65 [95%CI 1.11 - 2.47]	0.01	0.01
MESO	HR 0.77 [95%CI 0.36 - 1.66]	0.51	0.51	HR 1.46 [95%CI 0.73 - 2.89]	0.27	0.27
OV	HR 1.16 [95%CI 0.79 - 1.71]	0.42	0.42	HR 1.19 [95%CI 0.79 - 1.78]	0.39	0.39
PAAD	HR 1.07 [95%CI 0.61 - 1.89]	0.78	0.78	HR 0.84 [95%CI 0.46 - 1.53]	0.58	0.58
PCPG	HR 1.25 [95%CI 0.36 - 4.29]	0.71	0.71	HR 2.77 [95%CI 0.22 - 33.8]	0.4	0.42
PRAD	HR 0.71 [95%CI 0.40 - 1.26]	0.25	0.25	HR 0.62 [95%CI 0.10 - 3.78]	0.6	0.6
READ	HR 1.63 [95%CI 0.38 - 6.97]	0.49	0.5	HR 0.34 [95%CI 0.06 - 1.75]	0.17	0.2
SARC	HR 1.09 [95%CI 0.68 - 1.73]	0.71	0.71	HR 1.25 [95%CI 0.72 - 2.19]	0.41	0.42
SKCM	HR 0.72 [95%CI 0.52 - 1.00]	0.05	0.05	HR 0.72 [95%CI 0.49 - 1.05]	0.09	0.09
STAD	HR 1.29 [95%CI 0.78 - 2.12]	0.31	0.31	HR 1.09 [95%CI 0.70 - 1.69]	0.68	0.68
TGCT	HR 1.61 [95%CI 0.52 - 4.97]	0.4	0.4	N/A		
THCA	HR 1.81 [95%CI 0.77 - 4.25]	0.15	0.16	HR 6.60 [95%CI 0.83 - 52.4]	0.0243	0.073
UCEC	HR 0.82 [95%CI 0.36 - 1.88]	0.64	0.64	HR 0.98 [95%CI 0.32 - 2.93]	0.97	0.97
UCS	HR 0.70 [95%CI 0.26 - 1.83]	0.47	0.47	HR 1.04 [95%CI 0.38 - 2.80]	0.93	0.93
UVM	HR 2.47 [95%CI 0.82 - 7.47]	0.09	0.1	HR 3.98 [95%CI 1.06 - 14.9]	0.02	0.03

Supplementary Table 6: Genomic Test Result	
Category	Variant
Pathogenic Variants	*EWSR1-NFATc2* Fusion
	FANCE V311fs*2
Variants of Unknown Pathogenicity	*BCOR* A220S
	HIST1H1E A123G
	INHBA K272N
	MKI67 D2751N
	SMC3 S611N
Tumor Mutation Burden	3 Mutations per Megabase

Supplementary Figure 4: PET Scans – Case Report

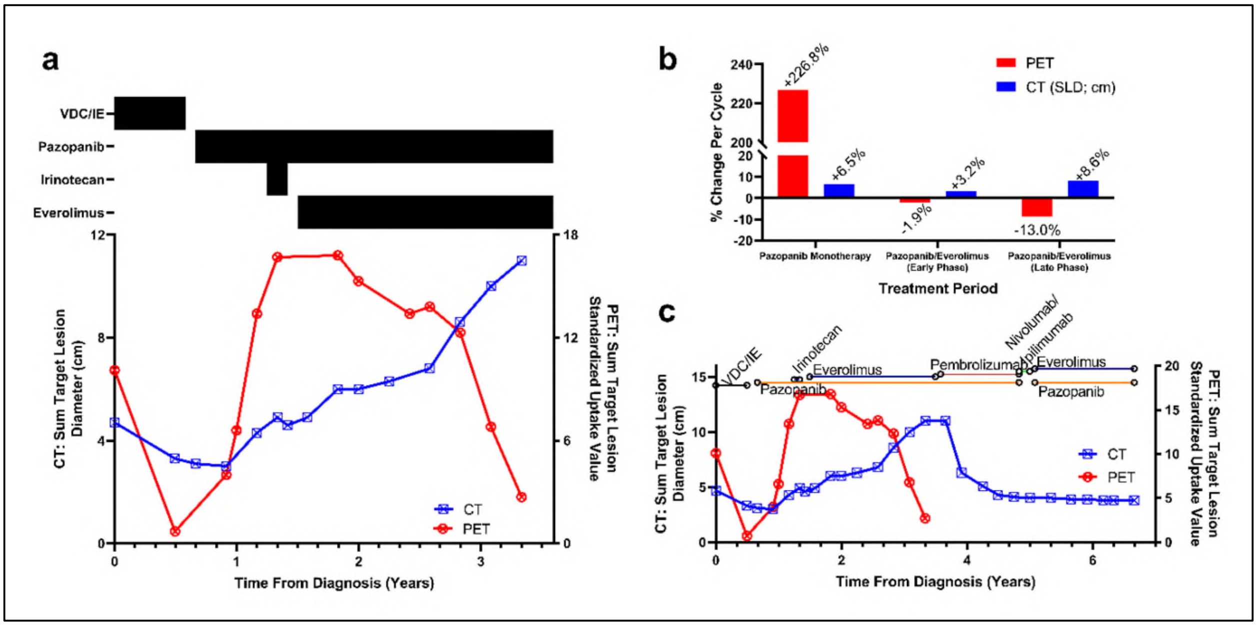

A 58-year-old man presenting with a 10-cm hypermetabolic mass and hyper-metabolic nodules was diagnosed with a high-grade, *EWSR1-NFATc2* fusion positive, small round-cell tumor. Surgical resection resulted in residual disease. **a**) Treatment course following initial resection until insurance denied further access to everolimus therapy and PET scans. **b**) Prior to the initiation of everolimus, target lesions steadily grew in size by both PET and CT scans. Initiating everolimus in combination with pazopanib resulted in modestly decreased growth rate measured by CT and a notable decrease in SUV as measured by PET. After an 18 months of everolimus in combination with pazopanib the growth rate of target lesions increased substantially by CT while SUV continued to decline by PET. **c**) Following the initial target lesions from baseline reveals a marked decrease in tumor size after treatment with immunotherapy. This resulted in stable indolent disease in the target lesions but did not take into account new lesions developed during immunotherapy. The patient was re-challenged with everolimus and pazopanib and continued on this therapy for 21 months without an increase in size of any lesion or the development of additional lesions. In total, the patient received 47 months of everolimus-based therapy.
